# Ovarian Dysgerminoma – Challenging Presurgical Diagnosis and Mini-Mally Invasive Treatment

**DOI:** 10.26502/acmcr.96550573

**Published:** 2023-02-03

**Authors:** K Otto, O Ebertz, C Matsingou, D Andrikos, RL De Wilde, H Krentel

**Affiliations:** 1 Clinic of Gynecology, Obstetrics, Gynecological Oncology and Senology, Acade-mic Teaching Hospital, Bethesda Hospital, Duisburg, Germany; 2 Clinic of Gynecology, Obstetrics and Gynecological Oncology, University Hospital for Gynecology, Pius-Hospital Oldenburg, Medical Campus University of Oldenburg, Germany

**Keywords:** Ovarian Dysgerminoma, Laparoscopy, Transvaginal Ultrasound

## Abstract

Dysgerminoma is a rare malignant germ cell tumor of the ovary that often affects women in reproductive age. The presurgical differentiation of dysgerminoma from benign conditions is challenging. In early stages, malignant dysgerminoma can be treated with fertility sparing surgery. We present a pictorial non-systematic review of literature, discuss diagnostic challenges in ultrasound and radiological imaging and present laparoscopic treatment options in a young woman with dysgerminoma.

## 1. Introduction

Dysgerminoma, one of the germ cell tumors of the ovary, is the most common malignant ovarian tumor in young women between the ages of 20 and 30. Germ cell tumors account for 2-3% of all ovarian malignant tumors [1-3]. Histologically it corresponds to the testicular seminoma in men [2]. It shows rapid growth, can become very large (diameter up to 50 cm) and is initially asymptomatic or causes rather non-specific symptoms as period irregularities and abdominal distension [2-4]. In advanced stages the tumors can be symptomatic with ovarian torsion, rupture, or hemorrhage [2]. The way of diagnosis is a pelvic examination with ultrasound and further imaging with MRI or CT [2]. 5% of dysgerminoma content syncythiothrophoblastic cells which produce HCG, which can be used as a tumor marker [5]. In 5-10% of cases they appear bilateral. In contrast to epithelial ovarian cancer, they are generally detected at early stages with a good prognosis [2,3]. The 10-year survival is about 90% [2,6]. While laparotomy is performed in early-stage epithelial ovarian cancer, there is a growing number of studies showing good results in terms of outcome for laparoscopic procedures in dysgerminoma regarding postoperative recovery and the recurrence free survival [7-9]. In the past, radical surgery via laparotomy with inspection of the pelvis, bilateral adnexectomy and total hysterectomy was performed [10], while today fertility-preserving therapy with tissue-sparing techniques of the contralateral adnexa in early stages has become established [1,2,10]. The prognosis is good even after less radical surgery [2,9,11,12]. In addition, most dysgerminomas are detected at stage I [2,3,9]. In stages beyond IA, after fertility-preserving therapy, adjuvant chemotherapy with cisplatin, etoposide, and bleomycin is recommended [2,13].

## 2. Methods and Material

We performed data research using pubmed and google scholar considering the keywords ovarian dysgerminoma, germ cell tumor, transvaginal ultrasound and laparoscopy, and present a case of dysgerminoma.

## 3. Results and Report of a Case

**Figure 1:**
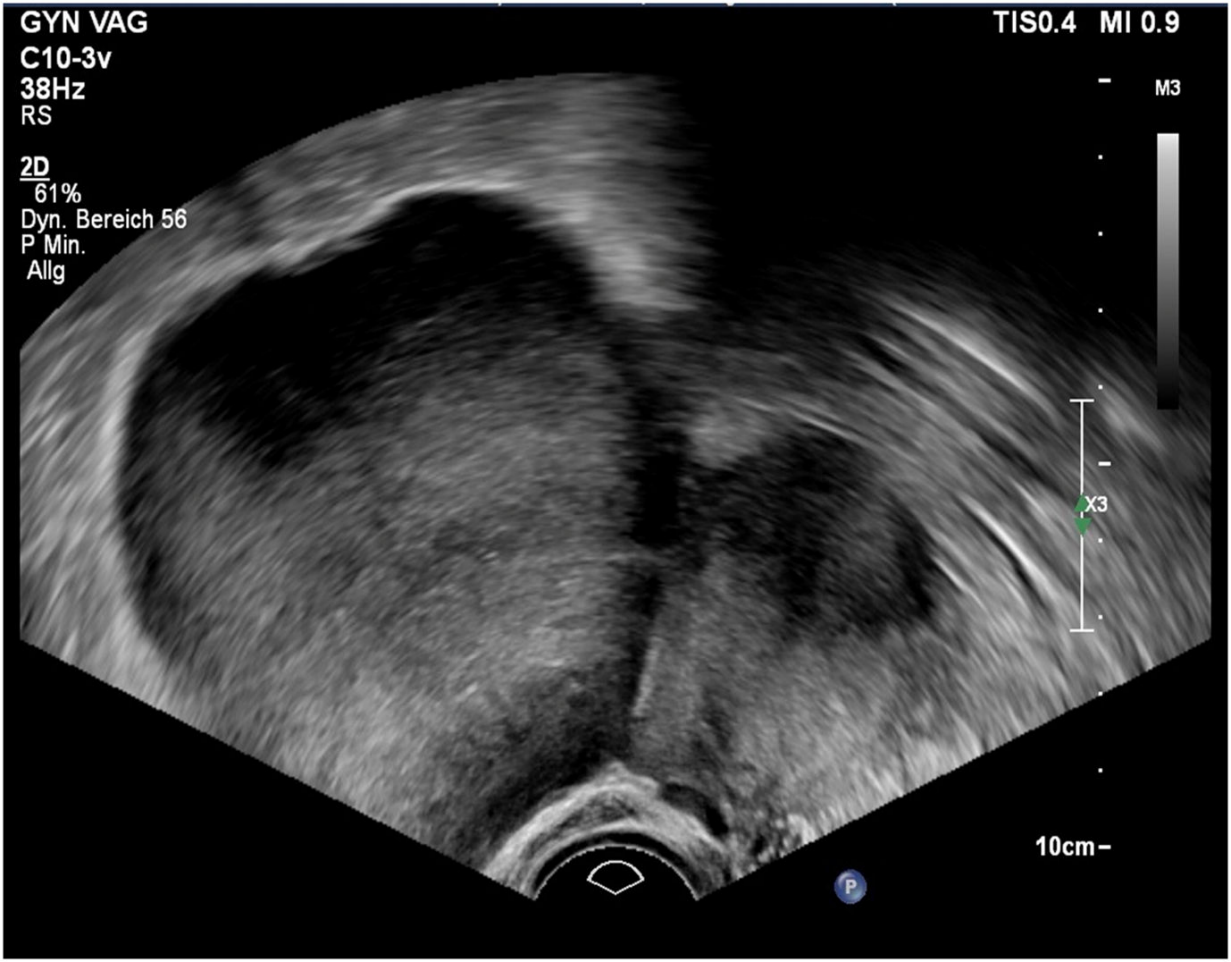
Transvaginal ultrasound image of dysgerminoma of left ovary (diameter 9,8 cm).

A 35-year-old IV-gravida IV-para has been referred to our cancer center for further investigation of an intraabdominal pelvic tumor of unknown origin. An externally performed MRI showed a 9 cm mass which the radiologists initially thought to be a subserous fibroid originating from the uterine wall. The initial symptom was lower abdominal pain. The patient presented with a history of four previous caesarean sections. Gynecological examination revealed a solid but mobile mass palpable in the pelvis. Transvaginal ultrasound showed a rather homogenous tumor with a diameter of 10 cm (Figure 1). There was no sign of irregular vascularization in additional doppler ultrasound. There was no sign of intraperitoneal aszites and the contralateral ovary showed no irregularities. At this time, we considered the tumor to be benign. In addition to the radiological report we tended to diagnose a uterine subserosal fibroid.

**Figure 2:**
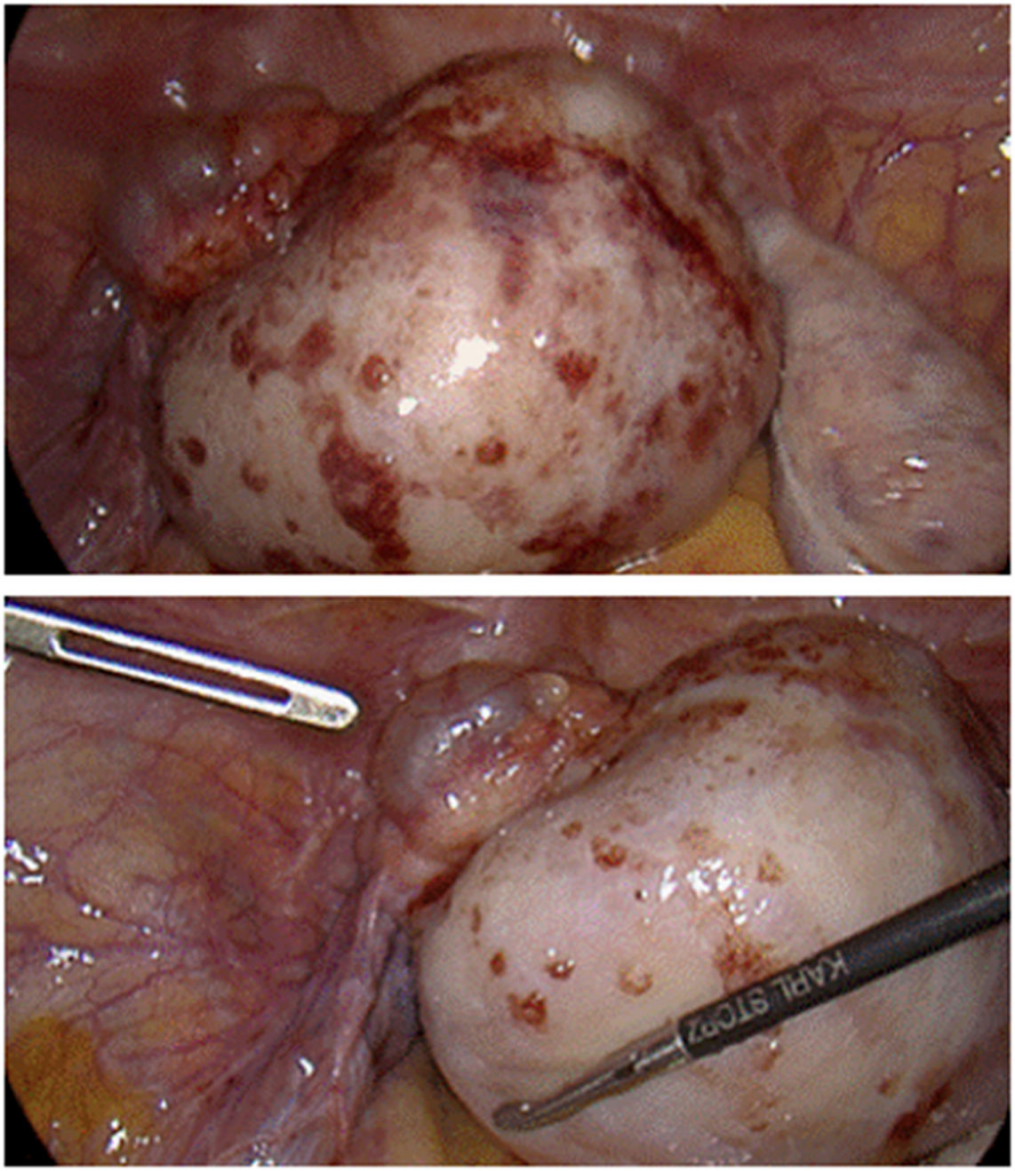
Laparoscopic view of dysgerminoma of left ovary.

We performed a diagnostic-operative laparoscopy which revealed a solid tumor originating from the left ovary (Figure 2). Uterus, right adnexa and pelvic peritoneum showed no signs of macroscopic pathologies. The ovarian tumor was completely excised with intact tumor capsule by partial oophorectomy and salpingectomy. Histological examination revealed the tumor to be a pT1a dysgerminoma according to TNM classification and FIGO stage IA. Immunohistological examination showed a negative reaction for CK7, CK20, calretinin, p40, WT1, synaptophysin and the estrogen receptor. Tumor cells were also negative for CK AE1 / AE3 + 5D3 and S 100, but showed a positive reaction for PLAP and CD117.

For further staging, computed tomography of the thorax, abdomen and pelvis was performed without any signs of metastasis. While CA 12-5 was slightly elevated, the tumor markers AFP, HCG and CA 15-3 were in a normal range. After diagnosing dysgerminoma, the patient was readmitted for surgical completion. Because of the patient’s young age, we performed a fertility sparing minimally invasive intervention by laparoscopy with complete adnexectomy of the left side, total salpingectomy on the right side, appendectomy, peritoneal washings, peritoneal samplings and infracolic omentectomy. Macroscopically and microscopically no residual tumor was diagnosed. Therefore, the final diagnosis was dysgerminoma of the left ovary, pT1a N0 M0 L0 V0 Pn0 R0, FIGO IA. The interdisciplinary oncological board decided on therapy-free aftercare. According to national guidelines, monthly clinical examinations with sonographic control and determination of the tumor markers AFP, HCG and CA 125 for six months after the operation [7,14] was recommended. The patient left the hospital in well-being 5 days after second surgery. At 6-month follow-up, there was no sign of recurrence. The patient was in a good general condition and had no complaints.

## 4. Discussion

The initial presurgical diagnosis of dysgerminoma is challenging as the condition is very rare and the imaging criteria are not clear. Malignant germ cell tumors usually manifest as rather inhomogeneous masses with both solid and cystic components [5]. To distinguish between malignant and benign ovarian tumors in transvaginal ultrasound, the IOTA criteria have been established [15] including features of possible malignancy and features of benign lesions resulting in a prognostic score. Features of benign ovarian tumors are a smaller size (diameter less than 4cm) and an entirely cystic component with no internal structure and lack of ascites. Signs of malignancy of ovarian lesions are a thick and irregular wall, solid and cystic components, thick septa, irregular vascularisation and ascites [5]. Suspicious ultrasound findings should be verified by radiological imaging techniques. Typical MR imaging findings in ovarian dysgerminoma are T2 - weighted prominent low intense fibromuscular septa in a usually large pelvic mass. Pelvic organ invasion, peritoneal implants and lymphadenopathy are possible additional findings [5]. However, in the presented case, transvaginal ultrasound and MR imaging showed a solid and homogeneous mass next to the uterus, without any additional findings. This was interpreted as a possible subserous uterine fibroid [16]. Moreover, it is also challenging to distinguish with certainty whether a tumor originates rather from the adnexa, the uterus or even intestine. A careful history of symptoms is the first step in differentiation. Tumor markers can give a further suspicion of origin and / or malignancy [17]. Additional examinations like colonoscopy, cystoscopy, gastroscopy can be performed in order to exclude gastrointestinal or urological tumors. We performed minimally invasive surgery as an additional diagnostic and therapeutical surgical approach. Laparoscopy revealed an ovarian tumor, but histopathological examination of the left ovary was needed in order to finally have a clear diagnosis. We decided to perform the secondary completing surgery also by laparoscopy. The advantages of minimally invasive surgery include less blood loss with less need for blood transfusions, fewer peri-operative complications, fewer risk for postsurgical adhesions, better cosmetic results, shorter stay in hospital and better recovery [7,18]. Previous studies already determined that early-stage malignant non-epithelial ovarian tumors can be removed laparoscopically with equally good outcome regarding complications, survival rate and recurrences compared to laparotomy [7-9]. Important limiting factors for laparoscopic surgery are the size of the tumor, the tumor stage, and the expertise of the operating surgeon [9]. Regarding the oncological safety and outcome, the intactness of the tumor capsule while excising the tumor is essential. Specific potential complications in laparoscopic treatment of oncological conditions are trocar metastasis, rupture of the tumor capsule and spilling of tumor cells [7,9,19]. Prado et al reported a case of recurrence of dysgerminoma after laparoscopic surgery at the site of tumor removal (cul-de-sac) [20]. To ensure that there is no carryover of tumor cells, recovery of the tumor should be done via mini-laparotomy or bag extraction [9]. Unilateral adnexectomy instead of excising the tumor has a lower risk of cell dissemination [21]. Infiltration of neighboring organs complicate or exclude a laparoscopic procedure. In contrast, in early ovarian epithelial cancer, the open approach is still recommended, because at present there are no randomized controlled trials of laparoscopic treatment for malignant epithelial ovarian tumors showing non-inferiority compared to open approach [21,22]. However, previous studies show a comparable survival rate when planned laparoscopic staging instead of laparotomy was performed in stage I epithelial ovarian cancer [23,24].

## 5. Conclusion

Presurgical diagnosis of ovarian dysgerminoma by imaging techniques is challenging. A combination of transvaginal ultrasound, MRI and computed tomography might help to presurgically detect a malignant dysgerminoma. Laparoscopic resection of the tumor or ovary with consecutive histopathological examination allows to differentiate dysgerminoma from other benign and malignant ovarian tumors. A two-stage laparoscopic surgery including fertility-sparing techniques with complete resection of tumor is the treatment approach of first choice in early-stage dysgerminoma.
